# Editorial: Molecular Imaging in Multiple Myeloma: An Update and Future Perspectives

**DOI:** 10.3389/fnume.2022.904502

**Published:** 2022-04-25

**Authors:** Antonia Dimitrakopoulou-Strauss, Christos Sachpekidis, Constantin Lapa

**Affiliations:** ^1^Clinical Cooperation Unit Nuclear Medicine, German Cancer Research Center, Heidelberg, Germany; ^2^Department of Nuclear Medicine, University of Augsburg, Augsburg, Germany

**Keywords:** multiple myeloma, PET/CT, PET-MRI, therapy diagnosis, therapy monitoring, prognosis

Multiple myeloma (MM) is the second most common hematologic malignancy after non-Hodgkin lymphoma accounting for approximately 1% of neoplastic diseases. MM is not curable despite the progress in diagnostic procedures and therapeutic approaches. Though still considered an incurable disease, its prognosis has been continuously improving since the ‘90s due to new therapies, including immunomodulators, proteasome inhibitors and monoclonal antibodies. Nowadays, MM can be classified as a chronic disease (1). In terms of imaging, modern diagnostic and staging modalities include computed tomography (CT), magnetic resonance imaging (MRI) and positron emission tomography/CT (PET/CT), mainly with the radiotracer ^18^F-FDG. ^18^F-FDG PET imaging has nowadays a great impact on the diagnostics and management of oncological patients and has gained increasing use worldwide (2). On the other hand, despite being a valuable tool in diagnosis, prognosis and therapy monitoring, ^18^F-FDG PET/CT has some limitations including a non-negligible rate of false-negative and false-positive findings in patients with MM (3, 4).

In this Research Topic Mesguich et al. present in an interesting review the strengths and limitations of both molecular hybrid imaging techniques, PET/CT and PET/MRI, for the diagnosis and therapy monitoring in MM. In particular, the role of ^18^F-FDG PET/CT in the diagnosis, staging, prognosis and monitoring of treatment response, also including new therapeutic protocols like immunomodulatory drugs and proteasome inhibitors, are discussed and compared to other imaging modalities, such as MRI and diffusion-weighted MRI (DW-MRI). Overall, DW-MRI has a high sensitivity for bone marrow involvement, whereas baseline ^18^F-FDG PET carries a strong prognostic value and a strong association with relapse risk and survival. Both imaging modalities ^18^F-FDG PET/CT and DW-MRI are crucial for the detection of extramedullary and paramedullary myeloma manifestations. Longitudinal ^18^F-FDG PET/CT studies are superior to MRI and DW-MRI for the evaluation of treatment response, since they provide an earlier assessment of post-therapeutic changes and prognosis with (even in the absence of standardized reading) a negative ^18^F-FDG PET/CT scan being associated with a longer PFS (Mesguich et al.). More studies are needed to address the role of novel therapies involving antibodies, antibody–drug conjugates, bispecific antibodies, and chimeric antigen receptor (CAR) T cells as mentioned by Mesguich et al. In the setting of response evaluation, both bone marrow-based minimal residual disease (MRD) diagnostics and ^18^F-FDG PET seem to have a complementary role meaning that double-negative patients demonstrate a longer PFS as compared to either MRD or ^18^F-FDG PET positive findings (Mesguich et al.) (5).

The role of several, non-^18^F-FDG radiopharmaceuticals is presented in another review by von Hinten et al. The authors discuss the impact of C-X-C motif chemokine receptor 4 (CXCR-4) imaging with ^68^Ga-Pentixafor, amino acid imaging with ^11^C-Methionine and ^11^C-Choline, proliferation imaging with ^18^F-FLT and imaging of bone remodeling with ^18^F-NaF. This review demonstrates the potential of Pentixafor also for theranostic approaches in MM using the combination of ^68^Ga-Pentixafor for selection of patients and ^177^Lu-Pentixafor for therapy (6). The value of labeled amino acids, in particular ^11^C-Methionine and the proliferation tracer ^18^F-FLT is topic of another review by Minamimoto. Both reviews demonstrate some advantages of these non-FDG tracers and show the complementary role to ^18^F-FDG, in particular in ^18^F-FDG-negative myeloma lesions. Main limitation of these non ^18^F-FDG tracers is the lack of studies in larger patient cohorts (7).

In conclusion, and contemplating the future of molecular imaging, the combination of fourth generation PET/CT scanners with extended field of view, which provide higher sensitivity, faster scanning protocols, application of less tracer activities, and more sophisticated reconstruction and image evaluation algorithms based on artificial intelligence approaches, will further improve diagnosis and therapy monitoring with PET/CT in MM (8). New dedicated tracers which may complement ^18^F-FDG in combination to MRD diagnostics, and/or sequencing of tumor probes, including whole genome sequencing, RNA sequencing, exome sequencing and gene expression, will provide a more holistic approach for the characterization of MM patients in the near future for both diagnosis and treatment response assessment and will allow personalized therapeutic approaches.

## Author Contributions

CL and CS: editing of manuscript. AD-S: writing of manuscript. All authors contributed to the article and approved the submitted version.

## Conflict of Interest

The authors declare that the research was conducted in the absence of any commercial or financial relationships that could be construed as a potential conflict of interest.

## Publisher's Note

All claims expressed in this article are solely those of the authors and do not necessarily represent those of their affiliated organizations, or those of the publisher, the editors and the reviewers. Any product that may be evaluated in this article, or claim that may be made by its manufacturer, is not guaranteed or endorsed by the publisher.
